# Mechanical Force Alters Morphogenetic Movements and Segmental Gene Expression Patterns during *Drosophila* Embryogenesis

**DOI:** 10.1371/journal.pone.0033089

**Published:** 2012-03-21

**Authors:** Abhishek Kumar, G. V. Shivashankar

**Affiliations:** 1 National Centre for Biological Sciences, Tata Institute of Fundamental Research, Bellary Road, Bangalore, India; 2 Mechanobiology Institute and Department of Biological Sciences, National University of Singapore, Singapore, Singapore; Centre for Genomic Regulation (CRG), Universitat Pompeu Fabra, Spain

## Abstract

The development of an organism is accompanied by various cellular morphogenetic movements, changes in cellular as well as nuclear morphology and transcription programs. Recent evidence suggests that intra and inter-cellular connections mediated by various adhesion proteins contribute to defining nuclear morphology. In addition, three dimensional organization of the cell nucleus regulate the transcription programs. However the link between cellular morphogenetic movements and its coupling to nuclear function in a developmental context is poorly understood. In this paper we use a point perturbation by tissue level laser ablation and sheet perturbation by application of force using magnetic tweezers to alter cellular morphogenetic movements and probe its impact on nuclear morphology and segmental gene expression patterns. Mechanical perturbations during blastoderm stage in a developing *Drosophila* embryo resulted in localized alterations in nuclear morphology and cellular movement. In addition, global defects in germ-band (GB) extension and retraction are observed when external force is applied during morphogenetic movements, suggesting a long-range physical coupling within the GB layer of cells. Further local application of force resulted in redistribution of non muscle myosin-II in the GB layer. Finally these perturbations lead to altered segmental gene (engrailed) expression patterns later during the development. Our observations suggest that there exists a tight regulation between nuclear morphology and cellular adhesive connections during morphogenetic movement of cells in the embryo. The observed spatial changes in patterning genes, with perturbation, highlight the importance of nuclear integrity to cellular movement in establishing gene expression program in a developmental system.

## Introduction

During early *Drosophila* embryogenesis, cells within an embryo are repositioned to different spatial regions in three dimensions and thus experience varied chemical gradients, established during cellular blastoderm stage by maternal proteins [1], [2], [3], [4]. This collective movement of cells is an essential step for the development of a multi-cellular organism [5], [6], [7]. In *Drosophila melanogaster*, GB invagination is one of the major morphogenetic movements for gastrulation, during which cells segregate into three germ layers - endoderm, mesoderm and ectoderm. At this stage, movement of cells is highly coordinated in both space and time and segmental gene expression patterns emerge [8], [9], [10]. These morphogenetic processes are also accompanied by changes in cellular morphology and possibly nuclear morphology. Differential inter-cellular contact and intra-cellular cytoskeletal reorganization are suggested as cellular mechanisms which lead to such large scale movement within the embryo [11], [12]. Force generated by apical localization of non-muscle myosin II in cells inside an embryo has been shown to be important for mesodermal invagination; ventral furrow formation with apical stabilization of myosin-II activated in response to active cell mechanical apexes oscillations [13], [14]. Several studies have identified maternal and zygotic genes required for proper invagination of cells and GB elongation [15]. Independent of nuclear shape changes, the role of cell shape has been demonstrated in modulating transcription program [16], activation of beta- catenin [17] as well as translocation of MAL-D [18]. Also, evidences suggest that concomitant with morphogenetic movements there are changes in cell shape [3], [11], [13], [15]. We propose that during morphogenetic movements there are changes in both cell shape and nuclear organization which might impinge on global transcription programs inside an organism. In this context, the link between nuclear morphology and the emergence of segmental gene expression pattern in a developing embryo is poorly understood.

In this work, we use micromanipulation methods in a live *Drosophila* embryo to elucidate the coupling between nuclear morphology, its position and global gene expression program. For this we have employed single point perturbation by tissue level laser ablation technique [19] and sheet perturbation by application of force using magnetic tweezers [20]. These perturbations lead to stalling of cellular movements - germ band extension (GBE) and retraction (GBR) suggesting that the mechanical coupling in GB cells may be necessary for collective movement inside live embryo. Further application of local force resulted in non muscle myosin-II redistribution in the cell layer and thus implicating that myosin II spatial localization is required for these morphogenetic movements. These defective movements are eventually reflected in altered spatial engrailed gene expression pattern in the embryo. In summary, we show that morphogenetic movements are highly coordinated and perturbations during this process lead to defects in GBE or GBR. Further the observed spatial changes in patterning genes with perturbation highlight the importance of nuclear integrity to cellular movement in establishing gene expression program in a developmental context.

## Results

### 1. Emergence of varied nuclear morphology during morphogenetic movements

**Figure 1:**
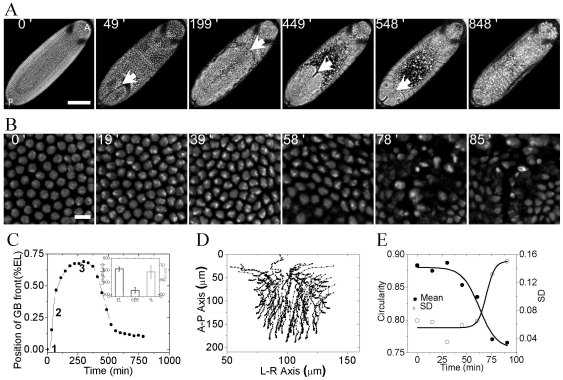
Nuclear morphology during morphogenetic movement- large scale collective movement of germ band positions cells in different regions of the embryo. (A) Maximum intensity z-projected confocal time lapse images of single live embryo showing movement of nuclei, marked by H2B-EGFP, at the dorsal side. White arrows indicate the position of germ band front at different times (Cellular blastoderm stage, during GBE, two-third extension of GBE, GBR, dorsal closure and segmented embryo respectively, corresponding time points are indicated at the top of each image). Scalebar = 100 µm. A-P denotes anterior and posterior axis of embryo. (B) Panel of images showing change in nuclear morphology starting from cellular blastoderm stage Scalebar = 10 µm. Region of interest (ROI) was chosen in the posterior half at the dorsal side of the embryo. (C) Typical plot of GB front displacement with time, measured from the posterior side. GB front moves towards the anterior side during elongation and comes towards the posterior during retraction leaving a sheet cells called amnioserosa (1, 2 and 3 are the positions when external perturbations are applied). Inset shows quantification of length of embryo and the extent of GB elongation measured (N = 32). EL = egg length (D) Two dimensional tracks of individual nuclei tracked during GBE measured by their centroid positions from Fig. 1A. Nuclei from posterior end were tracked. Direction of movement of cells is bottom to top. (E) Change in mean circularity and its standard deviation (SD) for nuclei at the dorsal side in a single embryo with time. 0′ in all these cases correspond to blastoderm stage.

In order to gain insight into changes in nuclear morphology during morphogenetic movements, confocal fluorescence imaging of a developing *Drosophila* embryo was carried out. Fig. 1A shows the movement of cells - GBE and GBR at the dorsal side of embryo, in which core histone-H2B is tagged with EGFP (Movie S1 - movie of embryo imaged at the dorsal side). There is collective movement of cells from ventral side towards the posterior end which further invaginate towards the anterior at the dorsal side. GBE is followed by GBR resulting in a segmented embryo. Arrows in Fig. 1A shows the movement of GB front with time. Fig. 1C shows a typical time trace of GB front movement. Post synctium, cellularization begins which is followed by GBE and the GB front reaches 2/3^rd^ egg length (measured from the posterior end, inset of Fig. 1C). During GBE, centroid positions of the H2B-EGFP nuclei were tracked till the nuclei moved out of the plane of focus; Fig. 1D shows typical tracks of nuclei in an embryo, (Movie S2 -movie of tracks of individual nuclei and Fig. S1A, movie of tracks was obtained from time lapse images using ImageJ plugin - MtrackJ). Minimal standard deviations in the nuclear displacement versus time plot of nuclei shows spatio-temporal coordination in the movement of cells during extension (Fig. S1B). Observed nuclear tracks suggest a sheet-like movement of cells in an embryo leading to collective coordinated movement. During the blastoderm stage, the nuclei are circular and concomitant with coordinated cellular movements, varied nuclear shapes emerge (Fig. 1B). At this stage, the two- dimensional cross-section of nuclei is highly circular over the whole embryo which changes shape as the embryo develops. Circularity, defined by 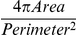, captures the emergence of heterogeneous nuclear shapes arising post cellularization (Fig. 1E). Time lapse images of nucleus (Fig. 1B and Fig. S2) also shows that initially post cellularization chromatin is homogeneous throughout but with development, heterogeneity in H2B intensity emerges suggesting alterations in chromatin compaction states within the nucleus [21]. The above results suggest that during development of an embryo, cells are positioned at destined locations during morphogenetic movements, which is accompanied by changes in nuclear shapes and size. In order to establish the importance of the above described morphogenetic movements to overall development of an embryo, we next used non invasive and non-genetic methods to perturb the dynamics of cell movement inside a live *Drosophila* embryo.

### 2. Local physical perturbation affects germ band extension and retraction

**Figure 2:**
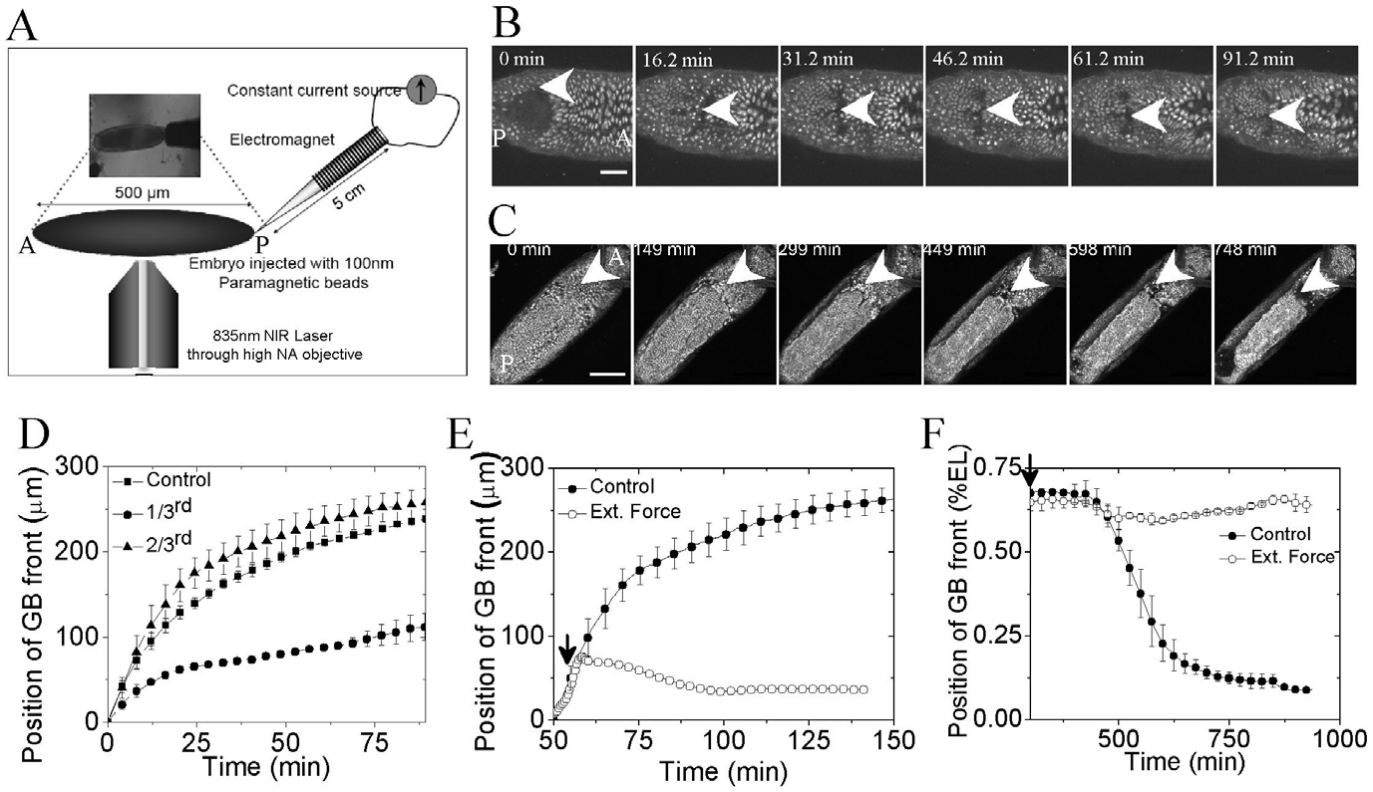
Perturbation techniques used and its effect on global movement of nuclei inside the embryo. (A) Schematic of perturbation technique used: Tissue level ablation at the dorsal side using 835 nm NIR (near infra red) multiphoton laser and application of external force (1.5 amp for 1.7 min) using a magnetic tweezers on embryo injected with 100 nm paramagnetic beads. (B) Time lapse images of GB front position post application of external force during GBE. Scalebar = 50 µm. 0 min' corresponds to start of GBE at the dorsal side. (C) Time lapse images of GB front position post application of external force post 2/3^rd^ extension of GB respectively. Scalebar = 100 µm. 0 min corresponds to time just before when GBR starts. White arrows in (B) and (C) indicate the position of GB front at times indicated on each image (D) Quantification of GBE, post ablation of a group of cells (15–20) at 1/3^rd^ 2/3^rd^ egg length and control embryo. (E) and (F) Comparison of GBE and GBR with the control embryos when force of applied during GBE (a typical plot) and post 2/3^rd^ extension of GB respectively (black arrows indicate times at which force is applied). N≥3. Error bars are standard deviations.

To probe the robustness of morphogenetic movement and its effect on *Drosophila* embryo development, two perturbation techniques were used: (1) Tissue level laser induced perturbation and, (2) Application of external force, using a custom built magnetic tweezers, on cells within an embryo, injected with 100 nm paramagnetic beads (schematic shown in Fig. 2A). Laser induced perturbation at a wider tissue level was used to establish if the GBE can be spatially modulated and to test if this altered GB position affects nuclear morphology. Embryos, post cellularization, were ablated at the dorsal side, 20 microns inside the embryo as measured from the chorion, at different positions along the egg length (EL) (schematic shown in Fig. S3). During GBE, as visualized from the dorsal side of the embryo, cells from the posterior side move towards the anterior till about 2/3^rd^ of egg length as measured from posterior end. Typically ∼750 ms duration ablation (∼190 mW at a spot) of 15–20 cells leads to large scale differential changes in GBE; depending on the ablation position (posterior end, 1/3^rd^ and 2/3^rd^ egg length). Fig. S4, time lapse images of cell movements, shows the dynamics of GBE post laser ablation. Movement of GB front, plotted in Fig. 2D, shows stalling of GBE below the ablation region in case of 1/3^rd^ and posterior end ablation. Nuclear trajectories were determined during GBE in control and 1/3^rd^ ablated embryos (control- Fig. 1D and Fig. S1A & 1/3^rd^ ablated- Fig. S5A). Trajectories of the groups of cells below the ablation spot are distinctly different from that above the ablation region, as observed from the nuclear displacement versus time graph (Fig. S5B). Cells which are below the ablated region (here, 1/3^rd^ egg length) do not move further towards the anterior region and thus get repositioned due to the perturbation. While ablation at 2/3^rd^ egg length does not have any effect on movement of cells inside the embryo. However ablation at posterior end stalls GBE and is lethal to the embryo, like that of 1/3^rd^ ablation. Further for 1/3^rd^ egg length ablation, nuclei of GB and early amnioserosa cells above the ablation point spread and increased in size, compared to those in a control embryo. Fig. S6A shows the representative images of nuclei in control and 1/3^rd^ ablated embryo and quantification of nuclear area is shown in Fig. S6B. These results suggest the impact of spatial positioning of cells in defining the nuclear morphology. Changes in nuclear morphology of cells above the ablation spot may also be attributed to decreased intercellular force exerted due to lack of GB movement.

Further, we used external force exerted on cells, coated with 100 nm paramagnetic beads, by using a magnetic tweezers to perturb morphogenetic movement inside developing *Drosophila* embryo (see Materials and Methods S1). 100 nm paramagnetic beads were injected during or before 11–13^th^ mitotic cycle of early development, before cellularization. Embryo was then allowed to develop at room temperature, leading to redistribution of microinjected beads and their nonspecific attachment. External force was applied from the posterior side by using an electromagnet mounted on xyz stage as shown in schematic Fig. 2A. Constant pulsed force was applied by fixing the current in the coil to 1.5 amperes for 1.7 minutes. The embryo was allowed to develop on the microscope, while time-lapse images were acquired. Embryos were perturbed at two different time points; during the fast phase of GBE and post 2/3^rd^ GBE. Application of pulsed force from the posterior end, during the fast phase of GBE, resulted in about 40 micron displacement of GB front accompanied by stalled GBE. Fig. 2B shows the time lapse images of the embryo post application of force. Fig. 2E compares the movement of GB front in the embryo which experienced the external force with that of control. Thus, morphogenetic movement inside an organism can be modulated by application of external force. Next, we applied the force when the GB had extended to 2/3^rd^ egg length. Similar force protocol was used as described above. Again in this case, the morphogenetic movement (GBR) is blocked as shown by comparing the GB front position in case of control and perturbed case (Fig. 2C- time lapse images and Fig. 2F- movement of GB front).

Our results suggest that cell-cell adhesion may be required for large scale movement of cells within an embryo. Perturbations either by using laser induced ablation or by stretching the cells resulted in altered cell movements within the embryo. These results demonstrate the role of physical perturbations on global positioning of cells within the embryo. In the next section, we probe the effect of force on nuclear morphology.

### 3. Force induced alterations in nuclear morphology during blastoderm

**Figure 3:**
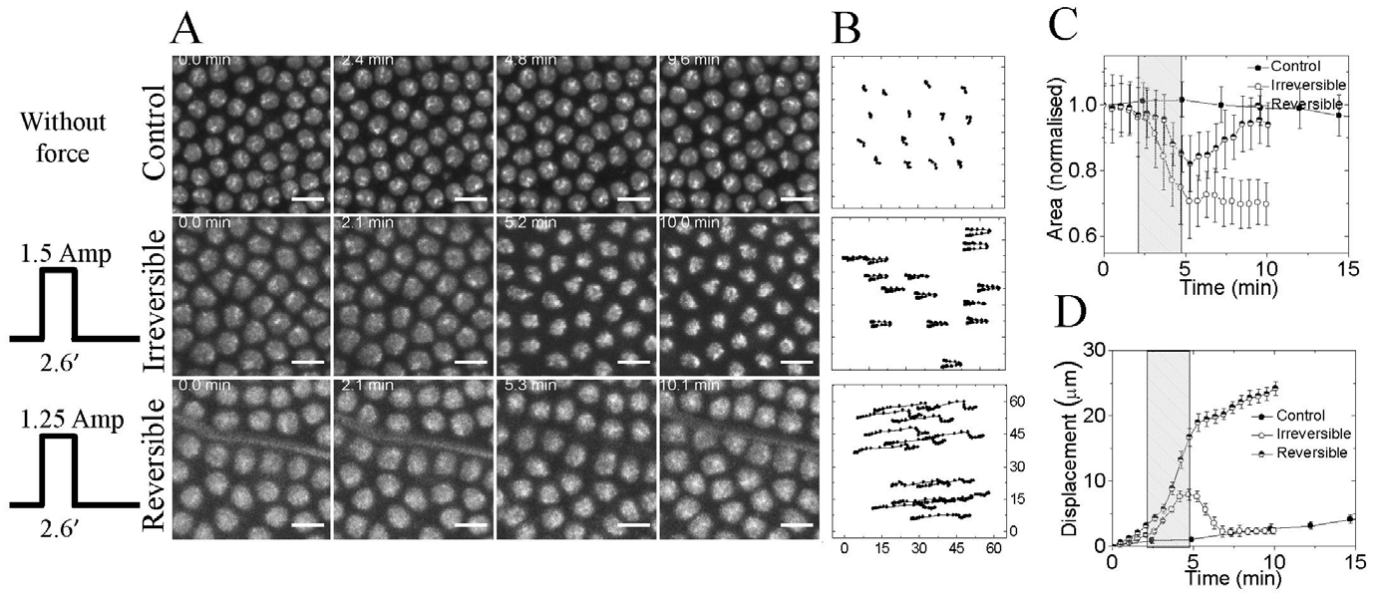
Reversible and irreversible modulation of nuclear size using force exerted by magnetic tweezers. (A) Panel shows the effect of application of external force (electromagnet was placed at the left side of the image) on nuclear size and (B) position (corresponding XY nuclear trajectory). Scalebar = 10 µm. (C) Normalized nuclear area is plotted with time (shaded region shows the time for which external force is applied). (D) Nuclear displacement with time on application of force is plotted indicating that external force displaces the nuclear position inside the embryo. For reversible case, N = 3; for irreversible, N = 4. ROI was chosen in the posterior half at the dorsal side of the embryo.

Application of external force, from the posterior side as before, elicited differential modulations in nuclear size, post cellularization at blastoderm stage (Fig. 3A, time lapse images for control, irreversible and reversible cases; corresponding movies- Movie S3, Movie S4 and Movie S5). The changes in nuclear size depended on amplitude of applied force. Higher force (using 1.5 ampere coil current, pulse duration 2.6 minutes) lead to approximately 30% shrinkage in nuclear size and was irreversible (Fig. 3C). On the other hand, application of slightly lesser force (using 1.25 ampere coil current, pulse duration 2.6 minutes) altered the nuclear size by ∼18% and on cessation of force, the nucleus reversed to its original size (Fig. 3C). In case of irreversible change in nuclear size, the embryo develops until 20 hr but stalls the emergence of larva. While the irreversible case is lethal to the embryo, reversible force does not hamper the growth of embryo. When compared with control, in which nuclear size changes by 10% over 30 minutes, application of force elicits about 30% change in nuclear size within 2.6 minutes (Fig. 3C). As the embryo develops, post cellularization, the dynamic cytoplasmic-nuclear links maintain the nucleus in a given morphological state. Application of force on cells leads to perturbations in cytoplasmic to nuclear links, thus eliciting changes in nuclear size. In this context, acto-myosin complexes have been shown to play an important role in maintaining nuclear shape. Apart from changing the nuclear size, force also displaces the cell from its original position by approximately 8 µ m (Fig. 3B, XY tracks of typical nuclei). We observe coordinated cellular movements when the force is applied during post cellularization of blastoderm stage. Force is propagated to at least a distance equal to one fourth the embryo size (120 µm). Nuclei at ∼80 µm and ∼130 µm, from the posterior, gets displaced by same length on application of force (Fig. 3D). In addition, the observed changes in nuclear size are similar till 130 µm from the posterior end. These results evidence distal propagation of force over large range, reaffirming physical connections between neighboring cells that get perturbed on application of force - resulting in altered morphogenetic movements. The induced local changes in nuclear size *in vivo* also alter the organization of chromatin, as seen from increase in H2B intensity on shrinkage. As stated earlier, non-muscle myosin II has been shown be important for cell movement and cell shape change [11], [22], [23]. Based on this, we next investigated the dynamics of myosin with development in control and perturbed embryos.

### 4. Transduction of local physical modulation to global defects during early embryogenesis is accompanied with myosin II relocalization

**Figure 4:**
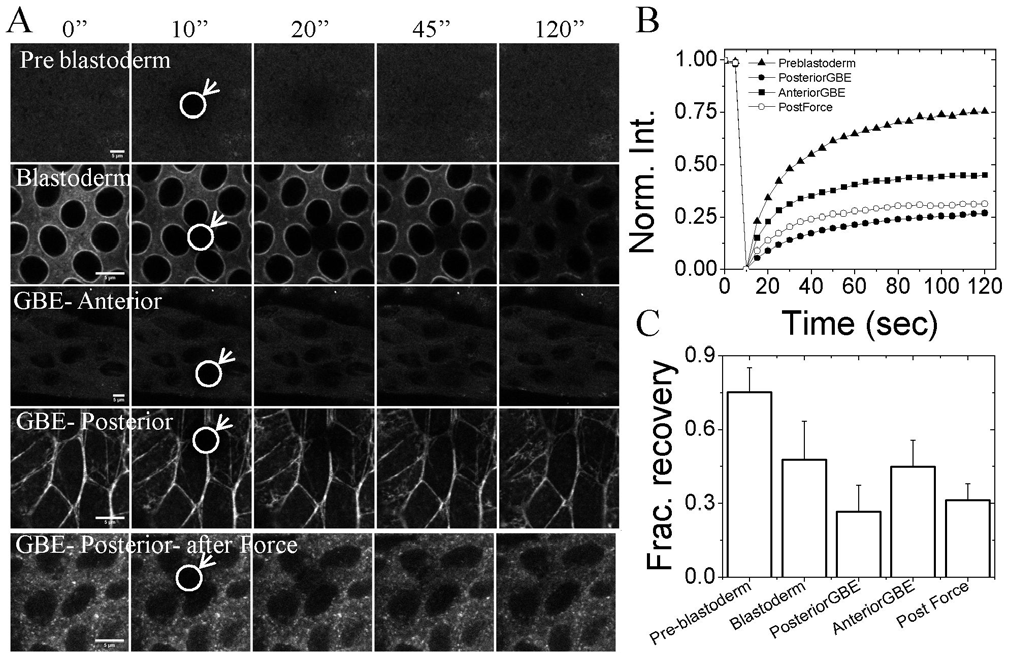
Localization and dynamics of non muscle myosin II during morphogenesis and on application of external force. (A) Panel shows the images pre bleaching 0″ and post bleaching- 10″, 20″, 45″ and 120″ at different stages of *Drosophila* embryo development as well as different regions in the embryo. First row: Pre-blastoderm stage (Bownes stage -4), second row: Blastoderm stage (Bownes stage -5), third row: anterior region during slow phase of GBE (early Bownes stage -9), fourth row: posterior region during slow phase of GBE (early Bownes stage -9). Circle and arrow highlights the region where photo-bleaching is performed. Scale bar = 5 µm (B) Plot shows the FRAP curves of EGFP tagged non muscle myosin II at different stages and regions inside a live embryo. (Filled Triangle: stage 4, Filled square: anterior stage 9, Filled circle: posterior stage 9 and open circle: posterior stage 9 after application of force) (C) Bar graphs show fractional recovery after 110 sec of bleaching in all the above cases. Curves and bar graph shown here are average of 7,13, 18, 27 and 8 curves for preblastoderm, blastoderm, GBE-anterior, GBE- posterior and GBE-posterior after force respectively. Single plane at the dorsal side was imaged for FRAP.

Dynamic reorganization of cytoskeletal networks begin, post 13^th^ mitotic cycle in the *Drosophila* embryo. During pre-blastoderm stage non-muscle myosin II is present in diffused form Fig. 4A, first row. However, post 13^th^ mitotic cycle myosin becomes more localized around the nucleus. Initially, during stage 5, it is apically present but as cellularization proceeds, it forms a ring around blastoderm nuclei Fig. 4A, second row. Myosin localization appears to be similar over the whole embryo with minimal heterogeneity between cells. With development, the localization of myosin becomes more heterogeneous. Cells which undergo long distant migration in the embryo have high localization of myosin, while cells which are less mobile evidence homogeneous distribution of myosin around the nucleus. Posterior cells show the presence of acto-myosin stress fibers which is required for cell migration. It is evident from Fig. 4A, third row that myosin is uniformly distributed in cells, anterior to GB front, which do not move much, while the cells in GB extending region show high localization of myosin at the inter-cellular junctions, Fig 4A, fourth row. Apart from the distribution of myosin, FRAP experiments reveal that the dynamics of myosin is spatially and temporally regulated in the embryo. During stage 4 of embryo development, myosin is freely diffusible, therefore, there is higher recovery post bleaching but with development and differentiation it becomes more localized and shows lesser recovery fraction, Fig. 4B and Fig. 4C. During stage 9, slow phase of GBE, anterior and posterior regions in the embryo show different dynamics of myosin. Cells which show large scale movement (posterior cells) in the embryo have high localization of myosin and less dynamic compared to cells (anterior cells) which show lesser movement, Fig. 4B. On application of force on posterior cells when GBE has just commenced, myosin becomes more diffuse and small punctas appear, suggesting that localization of myosin may be important for large scale cell migration (Fig. 4A fifth row) [14], [24]. Apart from these collective movements of cells and concomitant changes in nuclear shape as the embryo develops, transcription of various genes commences in a hierarchical manner. We next investigate the role of these perturbations on segmental gene expression patterns during embryo development.

### 5. Impact of altered nuclear morphology and morphogenetic defects on segmental gene expression pattern

**Figure 5:**
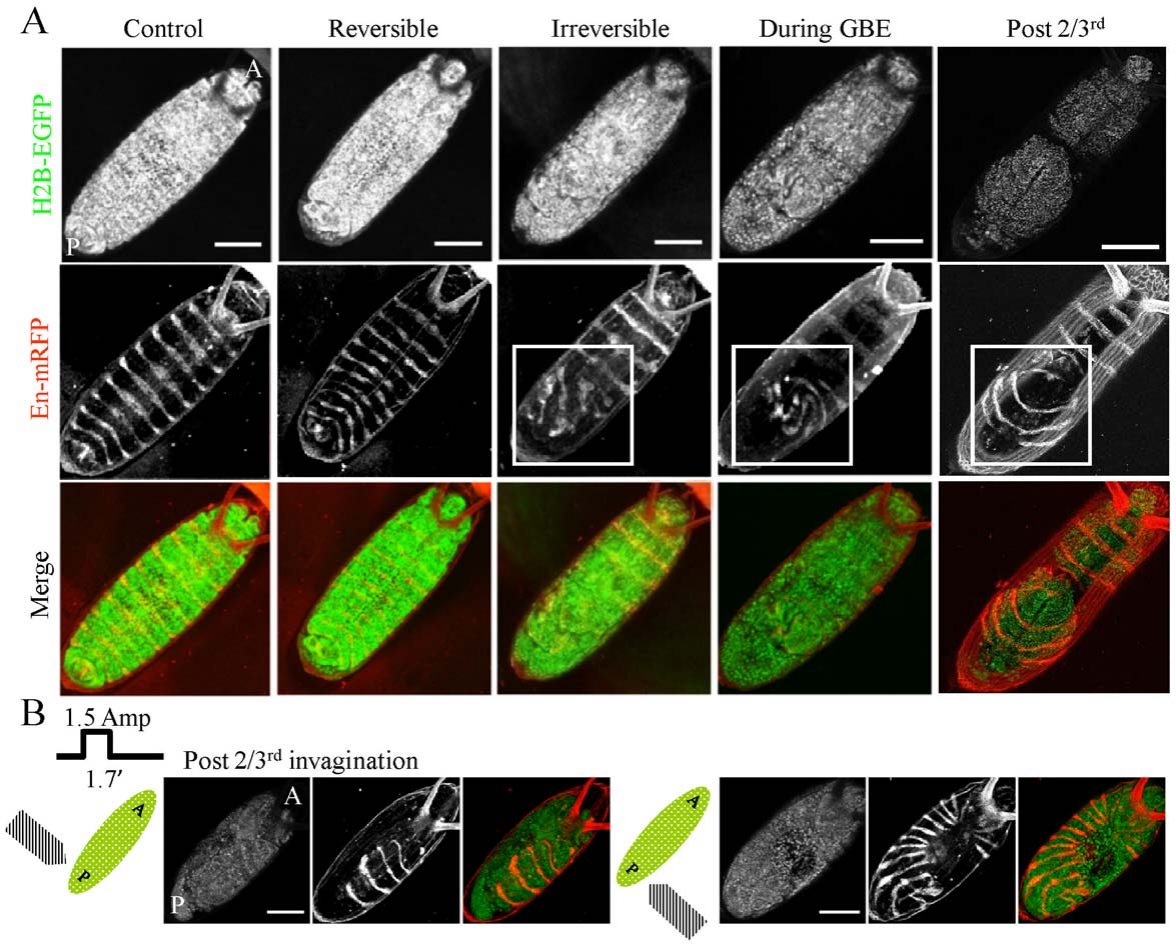
Effect of perturbation on engrailed expression and symmetry in patterning. (A) Panel shows the effect of application of external force during different stages of development from the posterior end on engrailed pattern 20 hour after egg laying. First row shows nuclear images marked by H2B tagged with EGFP. Second row is the engrailed pattern in the same embryo visualized by mRFP (;en-Gal4-UAS-myr-mRFP;). Third row is the merge of the above two. First column shows engrailed pattern in control embryo injected with 100 nm paramagnetic beads but without application of force while other columns show engrailed pattern post application of force during blastoderm stage (second and third column), during GBE (fourth column) and post 2/3^rd^ GBE (fifth column). For reversible case, N^+^/N^total^ = 17/21; irreversible case, N^+^/N^total^ = 76/89; during GBE, N^+^/N^total^ = 7/9; post 2/3^rd^, N^+^/N^total^ = 8/10. Scalebar = 100 µm. (B) Panel shows differential effect of application of force on engrailed patterning. Force is applied post 2/3^rd^ GBE and force protocol is as shown (1.5 amp for 1.7 min) either from right (N^+^/N^total^ = 4/7) or left (N^+^/N^total^ = 3/5) side of the embryo (corresponding schematic shown on left). All the embryos were imaged at the dorsal side and force was applied from the posterior side.

Next, to investigate the effect of such local perturbations during *Drosophila* embryo development, we mapped the expression of engrailed, a segment polarity gene [25]. For this, a transgenic expressing H2B-EGFP was crossed with recombinant expressing en-Gal4-UAS-myr-mRFP. As a control 100 nm paramagnetic beads were injected in these embryos as described earlier and the expression of engrailed was observed 20 hr post egg laying (Fig. 5A, first column- control images). Fig. S7 shows the emergence of engrailed pattern at different time points of *Drosophila* embryo development suggesting that with the microinjected beads but without the application of force there were negligible morphogenetic defects. As described before, when the force was applied during post cellularization of blastoderm stage, differential changes in nuclear size was observed; depending on the magnitude of applied force: reversible and irreversible change. In case of reversible changes in nuclear morphology, engrailed expression in all segments at 20 hr after egg laying was observed to be normal (Fig. 5A, second column) and all embryos hatched to larval stage. On the other hand, irreversible changes in nuclear morphology showed aberrant patterning of engrailed in the embryo (Fig. 5A, third column). Engrailed expression was normal in the anterior half while the posterior half, from where the force was applied, showed distorted patterns. In case of application of force during GBE, where the GB front stalled, also showed a similar phenotype of distortion of engrailed bands at the posterior side while the bands were intact at the anterior side (Fig. 5A, fourth column). Interestingly when the force was applied from the posterior end or from the two sides at the posterior end, after GB had extended to 2/3^rd^ position, revealed geometric defects in engrailed expression patterns. In these cases again, the patterns were distorted but distortion depends on the position from where the force was applied (Fig. 5A, fifth column – application of force from the posterior side, Fig. 5B, left – application of force from left side posterior end and right – application of force from right side posterior end). To assess the correlation between mechanical perturbations induced by microinjection and gene expression, we carried out both posterior and anterior injection of 100 nm paramagnetic beads. Fig. S8 shows that with no applied external force, the engrailed expression was similar to control in anterior and posterior injections. However, when the external force was applied from the posterior side (same as the irreversible force protocol in Fig. 3), the changes in engrailed expression pattern were similar suggesting that the anterior or the posterior injection exhibited identical behavior (Fig. S8). These results suggest that segmental gene expression patterns are sensitive to defects in morphogenetic movements and that there exists an inherent asymmetry in the movement of GB during retraction, which when perturbed by application of force, leads to distortions in engrailed expression pattern.

## Discussion

The cell fate map and gene expression patterns during cellular blastoderm in *Drosophila* embryos have been well characterized [26]. These stable gene expression patterns are a result of maternally derived morphogen signal gradients in the embryo [1], [3], [4], [27], [28], [29]. Morphogenetic cellular movements result in sensing these robust signaling gradients during early embryo development. Within the embryo, gene expression programs commences in a hierarchical manner - maternal mRNA, gap genes, segment genes and segment polarity genes. The mechanisms, which link morphogenetic movements to cellular and nuclear architecture and its impact on cell fate decisions during differentiation in developing embryos, is partially understood [16], [17], [18]. In addition, growing evidences emphasize the role of mechanical forces on gene expression programs during development [14], [16], [17], [18]. However, the link between chromatin conformation change due to mechanical strain and mechano-transduction of gene expression during development needs to be deciphered. Developing embryos therefore provide a good platform to spatially and temporally perturb morphogenetic movements and to study the coupling between geometric form and function.

The coordinated cellular movements during morphogenesis, evident from the cellular displacement versus time traces, show that cells are intercalated and cells migrate as a sheet during various morphogenetic movements, in our case GBE and GBR. Perturbations to this sheet, either due to ablation or application of external force, evidence differential changes in the coordinated cellular movement. Defects in GBE or GBR upon perturbation suggest that the developing embryos are highly sensitive mechanical systems. In addition, our results show that nuclear morphology is intimately linked with cellular movements; transiting from circular nuclear shapes to emergence of heterogeneity in nuclear architecture and chromatin compaction. This may be a result of cytoskeletal reorganization dynamics and due to changing inter-cellular adhesions with development. Our results further show that morphogenetic movements require localization of non-muscle myosin II at the inter-cellular junction and any perturbation to this localization, in terms of cytoskeletal integrity, leads to alterations in cellular movements within the embryo. From the force versus distance calibration plot shown in materials and methods S1, we estimate the ratio of force experienced by cells at the posterior end to that at one third egg length (Cell_one-third_/Cell_posterior_∼0.3). Cells near the electromagnet and further away did not show notably different displacement as shown in Fig. 3B. However, there was distal propagation of force that leads to tissue strain in the embryo. This force could deform the nucleus via the cytoplasm-nuclear connections or via the visco-elastic coupling or may just be the direct effect of force on distant cells as the thickness of the electromagnet is of the order of force propagation.

Changes in global expression patterns of segment polarity gene- engrailed, on application of force, may be both due to alterations in prestressed nuclear architecture and global repositioning of cells within the embryo. Further, application of force could also mechanically activate signaling pathways upstream of the engrailed, such as the Armadillo pathway, and lead to observed changes in gene expression [20], [30]. Myosin evidences differential dynamics in cells, depending on their morphogenetic movements suggesting that collective forces generated by intra-cellular acto-myosin complex may be important for morphogenetic movements and proper segmental gene expression pattern in the *Drosophila* embryo. The observed changes in segmental gene expression patterns, obtained when the force was applied from the two sides- left and right side of the embryo near the posterior end reveal a handedness in patterning genes or cellular movement. These results may have some bearing on earlier results, which showed that for correct handedness of embryonic hindgut in *Drosophila*, type-I myosin; Myo31DF was required [31], [32], [33]. In this case, the over-expression of type I myosin- Myo61F reversed the handedness in hindgut [33]. In response to mechanical strain, there have been observations of apical stabilization of myosin II under membrane in mesoderm at stage 6–7 [14] or into junction in the ectoderm at stage 9 post GBE [24] of *Drosophila* development respectively. Pouille *et al* [14] showed that intrinsic mechanical signal triggered myosin II redistribution and mesoderm invagination. In *snail* homozygous mutant where there is no mesoderm invagination, local mechanical deformation could rescue the cell movement by promoting Fog-dependent signaling leading to cortical sub-membranal myosin-II accumulation. Further, Fernandez-Gonzales *et al* [24] showed that myosin localization, at cortical junction, is tension dependent and is required for tissue elongation during *Drosophila* development. Our results further shows that application of force on posterior cells when GBE has just commenced leads to myosin II fluidification or formation of small punctas, suggesting that myosin reorganization was sensitive to mechanical tension. Taken together our results suggest that developing embryos comprise of a collective and dynamic mechanical network that exploits robust morphogen signaling gradients to elicit precise segmental genetic patterns. While the signaling gradients may be robust, our results highlight the importance of the mechanical sensitivity in physical cellular networks and their associated morphogenetic movements. Local perturbations to these morphogenetic movements are amplified to global defects, resulting in altered segmental gene expression patterns and thus developmental changes in the *Drosophila* embryo. However, quantitative links between physical modulation of cellular morphogenetic movements, acto-myosin reorganization and segmental gene expression patterns requires further investigation.

## Materials and Methods

### Fly stock and embryo preparation

Transgenic fly with EGFP (Enhanced Green Fluorescent Protein) fused to one of the core Histone protein, H2B, (H2B-EGFP) is used for labeling the nuclei in embryo. To see the global effect of perturbation on pattern formation en-Gal4 UAS myr mRFP recombinant was made by:

Step 1: ;en-Gal4; **(♀)** X ;UAS myr mRFP; **(♂)**
Step 2: ;en-Gal4/UAS myr mRFP; **(♀)** X ;Cyo/Tft; **(♂)**
Step 3: Screen larvae, grow till adult & collect Cyo ;en-Gal4 UAS myr mRFP/Cyo; **(♀)** X ;en-Gal4 UAS myr mRFP/Cyo; (**♂**)
Step 4: Cross used for experiment: H2B-EGFP **(♀)** X ;en-Gal4 UAS myr mRFP; **(♂)**

In these flies, H2B-EGFP marked the nucleus in green while the cells expressing the segment polarity gene- engrailed, had mRFP (monomeric Red Fluorescent Protein) at the plasma membrane. As a marker for cytoskeletal reorganization during embryo development (morphogenesis), transgenic fly with EGFP tagged to non-muscle myosin II regulatory light chain (MRLC) (spaghetti squash (Squash EGFP on chromosome II)) was used.

To collect freshly laid embryos, flies (males and females in ratio of approximately 1∶2) were kept in a cut bottle with sucrose plate at the bottom for an hour at 25°C in an incubator. Collected embryos were washed with water and aligned on #1 coverslip along a double sided tape with the dorsal side facing downwards and covered with Halocarbon oil 700 (Sigma, USA).

### Imaging and ablation

Experiments were carried out on either on Zeiss LSM 510 Meta confocal microscope using a 40×, 1.3 NA oil objective; 20×, 0.50 NA objective for whole embryo imaging and 63×, 1.4 NA oil objective for high resolution imaging (Carl Zeiss, Jena, Germany) or Olympus FV1000 confocal microscope using 60×, 1.4 NA oil objective and 20×, 0.70 NA objective (Olympus, Japan). Ablation experiments were performed using Titanium-sapphire 80 MHz pulsed femtosecond lasers (Tsunami or Mai-Tai – Spectra Physics, Mountain View, CA) mode locked at 835 nm. Embryos were aligned facing dorsal side down and ablation was carried out in one of the three regions at the dorsal side of the embryo: at the posterior end, 1/3^rd^ distance or 2/3^rd^ distance from the posterior end. In each case, ablation was carried out at blastoderm stage during the embryogenesis (see supplementary Fig. S3). The ablation was at the z plane where the nuclei had maximum projected area. This plane was selected by scanning the dorsal side of the embryo which has H2B EGFP expression. Ablation was carried out by parking 835 nm confocal spot to a diffraction limited region (∼190 mW) for 750 msec time duration using 40×, 1.3 NA objective (Carl Zeiss, Jena, Germany). Spot scan lead to the ablation of 15–20 cells. Post ablation, embryo was kept in a moist chamber at 25°C in an incubator to develop further.

### External force application and sample preparation

To apply mechanical force on cells in the embryo, early stage embryo, Bowne's stage 4 (11^th^ to 13^th^ mitotic cycle embryos) or before, were aligned on the coverslip as described above. 100 nm paramagnetic beads (micromod Partikeltechnologie GmbH, Germany) were injected in these embryos from the posterior side using Femtotips II (outer diameter 0.7 micron) on Eppendorf Femtojet microinjection set up. Embryos were allowed to develop till the required experimental stage at room temperature in a moist chamber. Force was applied using a custom made long pointed electromagnet mounted on a XYZ stage at 30° angle. Further details of electromagnet calibration and microinjection are provided in supplementary materials and methods S1. Post application of force, embryo was kept in a moist chamber at 25°C in an incubator to develop further.

### Fluorescence Recovery after Photobleaching (FRAP) experiment

FRAP experiments were performed on Olympus FV1000 confocal microscope using 60×, 1.4 NA oil objective (Olympus, Japan). All the experiments were acquired at 5 second per frame. For efficient bleaching, tornado bleach option was used after second frame with 100% 488 nm line of Argon ion laser (Melles-Griot Laser Group, Carlsbad, CA) for 50 iterations with pixel time 8 microsecond in a circular region of interest (ROI) with diameter 90 pixel (approximately 3.7 micron). Post bleaching 23 additional frames was acquired. Background subtraction was done during analysis to correct for photobleaching and nuclear movements were also corrected.

### Image analysis and quantification

Acquired images were processed and analysed using ImageJ software (http://rsbweb.nih.gov/ij/index.html), Zeiss LSM image examiner and Olympus FV10 ASW. Nuclear tracks were determined by using ImageJ plugin- MtrackJ (http://www.imagescience.org/meijering/software/mtrackj/). All the graphs and quantifications were done using OriginPro 7.5 (OriginLab Corporation, Northampton, USA).

## Supporting Information

Figure S1 **Coordinated movement of nuclei during GBE.** (A) XY tracks of the centroid position of individual nuclei (marked by H2B-EGFP) are determined by tracking them using ImageJ plugin (MtrackJ) from the time lapse images acquired (B) Displacement versus time plot shows highly coordinated movement of nuclei during the morphogenetic movement (position 0 shows the starting point of individual nuclei at time 0 min (post cellularization of Blastodem stage). Nuclei from posterior end were tracked. Direction of movement of cells is bottom to top (A-P axis).
(TIF)

Figure S2 **The evolution of nuclear shape in a** ***Drosophila*** **embryo.** Note the spherical nuclei at the earlier time points: more asymmetric shapes emerge with cellularization, and shapes become more variegated and region specific with the onset of germ band extension. The representative z-stack projected images shown here start from just before 13^th^ mitotic cycle (0′) and continue into germ band extension, 112′ later at room temperature. The time points in minutes are indicated. Dorsal side of the embryo is imaged. Scalebar = 20 µm.
(TIF)

Figure S3 **Schematic details of laser induced perturbation experiment.** Image shows the dorsal view of a live embryo expressing H2B-EGFP. Markings on arrow indicate different positions of ablation – posterior end, 1/3^rd^ and 2/3^rd^ from the posterior. Ablation is performed at one of the three regions using Titanium sapphire multiphoton laser mode locked at 835 nm optimized to give 190 mW at the focal plane of 40×, 1.3 NA objectives.
(TIF)

Figure S4 **Effect of tissue level ablation on GBE.** Each row shows the movement of cells and GBE front in control, 2/3^rd^ ablation, 1/3^rd^ ablation and posterior end ablation respectively at different time points (0′, 20′, 40′, 60′, 80′ and 100′) after ablation. 0′ corresponds to cellular blastoderm stage. The plane in which most nuclei are observed was chosen to be the ablation plane (dorsal side). For spot ablation, 835 nm laser was parked at a diffraction-limited region for ∼750 msec at one of the three positions as described. The images shown above are 3D reconstruction image using Zeiss LSM 510 Meta software. Scalebar = 50 µm.
(TIF)

Figure S5 **Arrest of GBE by tissue level ablation of** ***Drosophila*** **embryo.** Nuclear XY trajectories for the embryo ablated at 1/3^rd^ position from the posterior end obtained from the time lapse images. (A) XY tracks of 70 nuclei determined using ImageJ plugin- MtrackJ. 2D tracking is done till the nuclei went out of the plane. Ablation perturbs the movement of nuclei as seen from the tracks and also stops the GBE below the ablation point. (B) Movement of cells below and above the ablations spot.
(TIF)

Figure S6 **Impact of spatial position on nuclear size.** (A) Representative images (Scalebar = 25 µm) and Nuclear area of the cells in front of GB front (early amnioserosa cells) is plotted for the control and 1/3^rd^ ablated after 2.5 hr of cellularisation. N = 4. (All error-bars are standard deviations, * implies p<0.005).
(TIF)

Figure S7 **Engrailed pattern in embryos at different stages of development.** First column shows the nuclei image as marked by H2B-EGFP, second column shows engrailed pattern and third is the merge. Embryos are imaged post GBE (first row), post GBR (second row) and 20 hour after egg laying (third row).
(TIF)

Figure S8 **Engrailed patterning in embryo injected with 100 nm paramagnetic beads from the posterior and without force & with force, anterior region without force application and with irreversible force application protocol (as described in main manuscript).**
(TIF)

Materials and Methods S1 **Calibration of electromagnet to estimate the force exerted.**
(DOC)

Movie S1 **Movie of morphogenetic movement namely germ band extension (GBE) and retraction (GBR) in a live embryo. Nucleus labeled with H2B-EGFP.**
(AVI)

Movie S2 **Movie of tracks of individual nuclei in a live embryo during cell movement at the posterior region.**
(AVI)

Movie S3 **Movie for control case showing zoomed in view of nucleus labeled using H2B-EGFP, when force was applied during the blastoderm stage in embryos injected with 100 nm paramagnetic beads.**
(AVI)

Movie S4 **Movie for irreversible case showing zoomed in view of nucleus labeled using H2B-EGFP, when force was applied during the blastoderm stage in embryos injected with 100 nm paramagnetic beads.**
(AVI)

Movie S5 **Movie for reversible case showing zoomed in view of nucleus labeled using H2B-EGFP, when force was applied during the blastoderm stage in embryos injected with 100 nm paramagnetic beads.**
(AVI)
