# Supplementary figures and images for: Mechanical Force Alters Morphogenetic Movements and Segmental Gene Expression Patterns during Drosophila Embryogenesis
Source: PLoS One. 2012 Mar 21;7(3):e33089. doi: 10.1371/journal.pone.0033089 (PMC3310051; doi:10.1371/journal.pone.0033089)

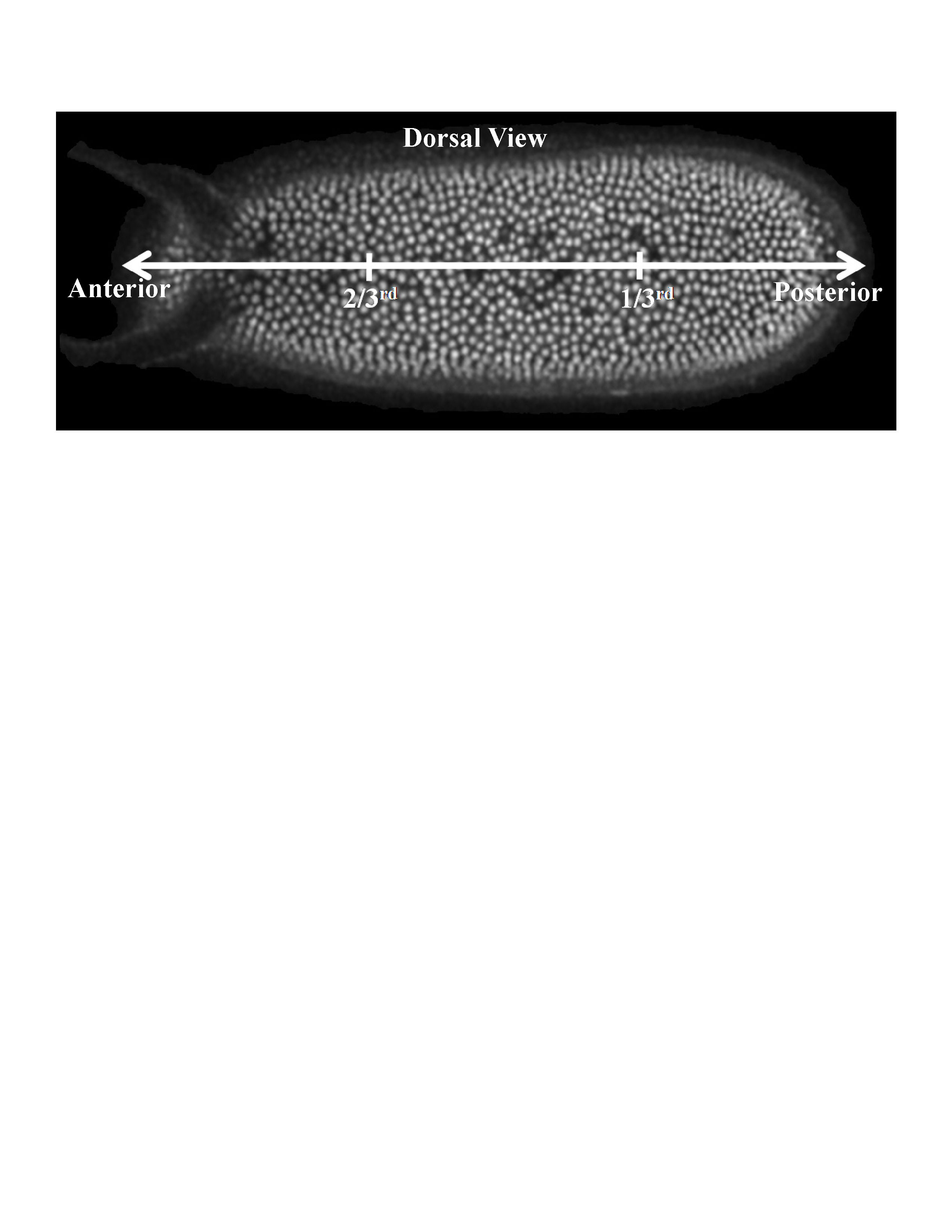

Supplement: Figure S3 — Schematic details of laser induced perturbation experiment. Image shows the dorsal view of a live embryo expressing H2B-EGFP. Markings on arrow indicate different positions of ablation – posterior end, 1/3rd and 2/3rd from the posterior. Ablation is performed at one of the three regions using Titanium sapphire multiphoton laser mode locked at 835 nm optimized to give 190 mW at the focal plane of 40×, 1.3 NA objectives. (TIF) [file pone.0033089.s003.tif]

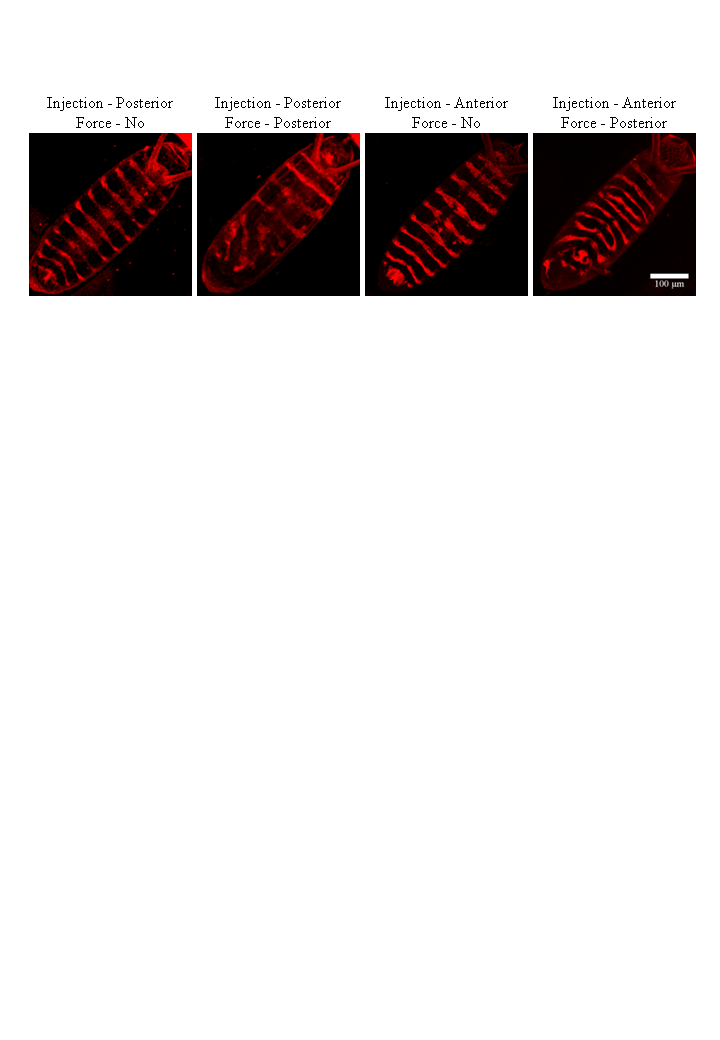

Supplement: Figure S8 — Engrailed patterning in embryo injected with 100 nm paramagnetic beads from the posterior and without force & with force, anterior region without force application and with irreversible force application protocol (as described in main manuscript). (TIF) [file pone.0033089.s008.tif]
