# Supplementary material for: Mechanical Force Alters Morphogenetic Movements and Segmental Gene Expression Patterns during Drosophila Embryogenesis
Source: PLoS One. 2012 Mar 21;7(3):e33089. doi: 10.1371/journal.pone.0033089 (PMC3310051; doi:10.1371/journal.pone.0033089)
Supplement: Materials and Methods S1 — Calibration of electromagnet to estimate the force exerted. (DOC) [file pone.0033089.s009.doc]

**Materials and Methods S1: Calibration of electromagnet to estimate the force exerted**

Sub-resolution beads (100nm) non-fluorescent paramagnetic beads were microinjected inside the live *Drosophila* embryo during (or before) Bowne’s stage 4 (11^th^ to 13^th^ mitotic cycle embryos) from the posterior end. Earliest time of force application was cellular blastoderm stage, by this time these beads can diffuse across in the embryo. Mitotic divisions and diffusion can distribute the small bead uniformly across the length of embryo. The beads could not be visualized inside the embryo under a DIC microscope due to high scattering by the yolk. Since beads were injected pre-cellularisation and there is subsequent mitosis, beads could be in the cytoplasm as well as inside the nucleus.

The density of beads injected was 7*10^12^ beads per ml. Considering the volume of beads injected (5% of total volume of embryo = ~0.52nl), we estimate the total number of beads injected in the embryo to be 3.64*10^6^. The electromagnet used for the force application was calibrated using 4µm paramagnetic beads suspended in 96% glycerol solution. Force calibration was carried out for different amplitude of current in the electromagnet (0.5, 1.0 and 1.5 amps). Shown below is the plot of force exerted on the bead as a function of distance from the electromagnet.


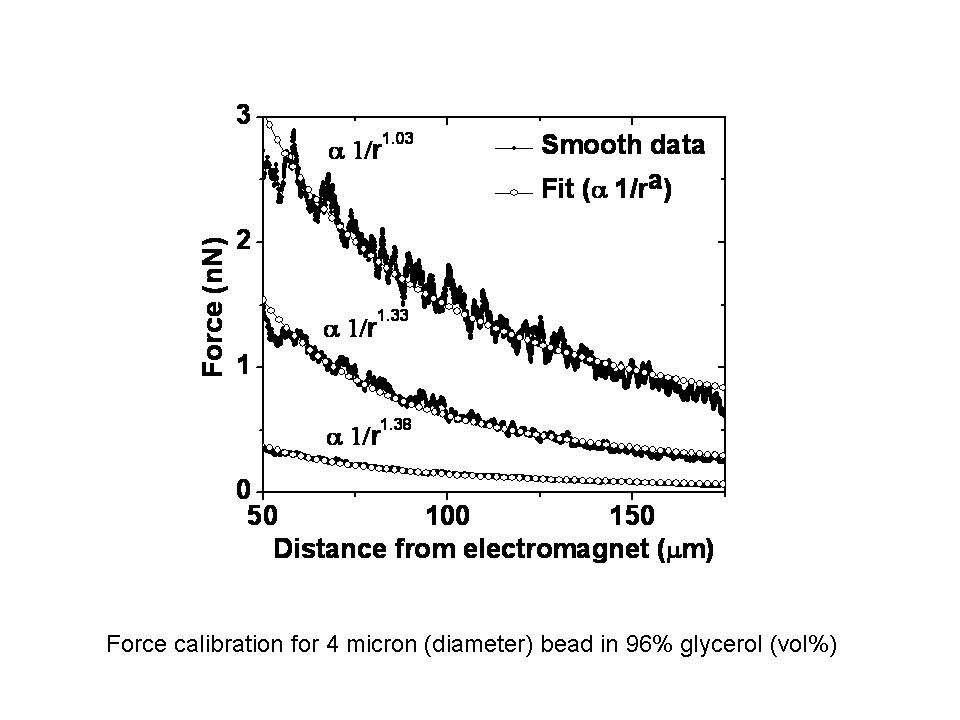


In the experiment 100nm paramagnetic beads were used. Also the magnetization property of the two beads were different (m_4μm_= 3.0 emu/g particles, 2.7*10^7^ particles per mg; m_100nm_= 24 emu/g particles, 1.5*10^12^ particles per mg). Therefore, we need to scale this graph taking into account the magnetization of the two beads. Using,
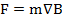
, we get, F_100nm_= (m_100nm_/ m_4μm_)*F_4μm_. Hence, force due to 100nm bead is 0.43pN at 50 μm from electromagnet. Assuming all the beads injected beads attach to 6000 cells during blastoderm stage, effective force experienced is 0.26nN (sub nanoNewton force).
